# Simple *in vitro* single-stranded linear and circular DNA preparation, functional selection, and validation using phosphor-derived modifications

**DOI:** 10.1016/j.jbc.2025.110874

**Published:** 2025-10-29

**Authors:** Seyed Vahid Hamidi, Vanessa Aguilar-Sánchez, Vincent L. Héroux, Jonathan Perreault

**Affiliations:** 1 INRS Centre Armand-Frappier Santé Biotechnologie, Laval, Quebec, Canada; 2 Department of Bioengineering, McGill University, Montreal, Quebec, Canada

**Keywords:** circular ssDNA, phosphorothioate modification, single-stranded PCR product, lambda exonuclease, SELEX

## Abstract

Interest in the preparation of circular ssDNA library has been increasing recently; therefore, developing a simple and an efficient method for circular DNA generation will be very useful for all procedures and techniques that are dependent on circular ssDNA preparation. In this study, a new simple method for *in vitro* preparation of circular ssDNA is proposed. We hypothesized that using a phosphorylated—phosphorothioated primer would not affect the efficiency of PCRs but, more importantly, would suppress the activity of the lambda exonuclease enzyme even if it is phosphorylated. The produced phosphorylated ssDNA is ready to be circularized *via* a ligation reaction using a bridging oligonucleotide. Several optimizations and enhancements have been conducted in the ligation reaction, notably by embedding an extra thymine nucleotide at the ligation site to compensate for the additional adenosine nucleotide added by Taq during the PCR. In addition, the performance of the proposed method has been validated by selecting linear and circular aptamers against Middle East respiratory syndrome coronavirus spike protein during 15 successive cycles of SELEX. Because this new method is simple and user friendly, it has a potential to be automated for high-throughput purposes and may further stir growing interests in preparation of circular ssDNA and its applications.

Due to the broad applications of *in vitro* ssDNA preparation in molecular biology, numerous techniques have been developed for this purpose. So far, ssDNA preparation methods have been utilized for systematic evolution of ligands by exponential enrichment (SELEX) (1), oligonucleotide microarray preparation (2), PCR-based padlock probe (PLP) generation (3), circular aptamer selection (4, 5, 6, 7, 8, 9, 10, 11, 12, 13, 14, 15, 16, 17, 18), single-stranded–labeled probe production (19), and coupled PCR–rolling circle amplification (RCA) for gene production (20). In addition, it has been used for linear and circular ssDNA library generation for DNA sequencing (21), production of origami nanostructures (22), as well as long ssDNA donors for genome editing using CRISPR (23, 24, 25, 26). A good example of the use of ssDNA is aptamers. Aptamers are small ssDNA or RNA molecules that fold into well-defined three-dimensional structures with a high affinity and specificity for their target molecules (27). Also, they have better thermal stability, lower cost, and are easier to modify compared with antibodies. They are selected by SELEX procedure, which starts with a random oligonucleotide library (*e.g.*, 10^15^ sequences) followed by four key steps: (i) specific binding of oligonucleotides (aptamer) with the target, (ii) extraction of the bound oligonucleotides, (iii) amplification of the extracted sequences with PCR, and (iv) production of an enriched pool of single-stranded aptamer sequences that will be used again in step (i), and so on. After completion of the SELEX procedure, following ∼10 iterations of the four steps, the candidate aptamers are sequenced for characterization (28). However, developing a simple, fast, efficient, and user-friendly technique to produce pure ssDNA is still challenging (29). The majority of ssDNA-producing procedures, such as denaturing PAGE and biotin–streptavidin–based techniques, are dependent on dsDNA denaturation and detachment of the sequence of interest from the complementary strand. Denaturing PAGE has been frequently utilized for pure ssDNA preparation; however, it is not convenient for high-throughput approaches (29, 30, 31). Asymmetric PCR and biotin–streptavidin–based methods are also criticized because of dsDNA impurity byproducts that are generated during the process (19, 29, 32, 33, 34). On the other hand, enzymatic degradation of the undesired strand *via* lambda exonuclease enzyme is a simple and robust way to produce pure ssDNA. It is reported that this enzyme has 20 folds more activity on 5′-phosphorylated strands of dsDNA than on hydroxylated strands, causing the hydroxylated strands to get single stranded following digestion (32, 34, 35), but in principle, this also precludes its use for any application that requires the presence of a 5′-phosphate, such as preparation of PLPs and circular DNA.

The effect of chemically modified nucleotides resistant to nucleases, including phosphorothioate bond modifications for ssDNA generation, has already been investigated (36, 37). In this study, phosphor-derived modifications have been used for *in vitro* production of ssDNA, linear or circular depending on the application. For this purpose, PCR was performed using phosphorylated–phosphorothioated forward primer and phosphorylated reverse primer using a 100-base template (Fig. 1, *A* and *B*). In the current work, we show that although the lambda exonuclease enzyme has robust digestion activity on phosphorylated strands of dsDNA, its degradation activity is completely suppressed on phosphorothioate-modified strand, even if it enjoys 5′ phosphorylation modifications (Fig. 1*C*). In addition, the produced phosphorylated ssDNA can be circularized using complementary strands without prior phosphorylation steps (Fig. 1, *D*–*F*). Therefore, the whole procedure of circular ssDNA preparation has become much faster and simpler. In addition, an increased DNA ligation efficiency using T4 DNA ligase was obtained by embedding an additional thymidine nucleotide exactly at the ligation site to account for the adenine added by Taq during PCR. This simple, fast, and robust method will be useful for specific ssDNA applications and is a good candidate for automation purposes because of the broad application of ssDNA preparation in molecular biology. In addition, this technique has been successfully applied on SELEX for linear and circular aptamer selection against Middle East respiratory syndrome coronavirus (MERS-CoV) spike protein.

**Figure 1:**
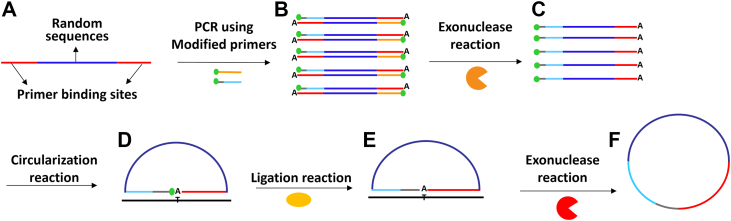
**PCR product circularization strategy.***A*, schematic representation of the template used with specific sequences as PCR primer–binding sites (*red line*) at the extremities and random sequences (*dark blue line*) in the *middle*. *B*, PCR using reverse primer (*orange line*) with 5′ phosphorylation modification (*green dot*) and forward primer (*light blue line*) with 5′ phosphorylation modification (*green dot*) and five phosphorothioate bonds (*gray line*) at the far 5′ end of the primer. The “A” illustrates the adenine added by the Taq DNA polymerase at the 3′ end of its product. *C*, exonuclease reaction by utilizing the lambda exonuclease enzyme (*orange semicircle*) for degradation of the nonphosphorothioated reverse strand. *D*, single-stranded PCR product (ssPCRP) circularization *via* complementary strand (*black line*). *E*, head-to-tail sealing of ssPCRP through ligation reaction using a ligase enzyme (*yellow ellipse*). *F*, finally, nonligated ssPCRP and the complementary strand are degraded by the action of exonuclease I enzyme (*red semicircle*).

## Results

### PCR optimization using modified primers

**Figure 2:**
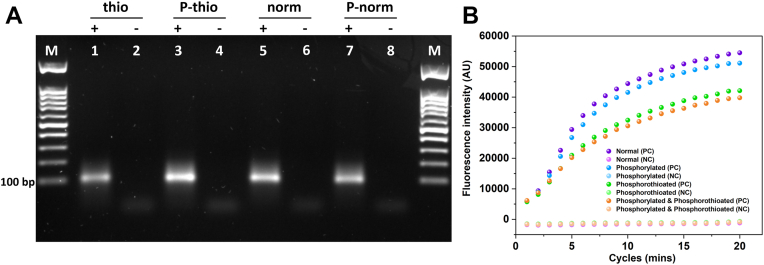
**PCRs using modified primers.***A*, lane M: markers; lanes 1, 3, 5, and 7: positive reactions (with template); lanes 2, 4, 6, and 8: negative reactions (without template). All PCRs have been done by using a phosphorylated reverse primer (1 μM). Lanes 1 and 2: PCR with phosphorothioated forward primer. Lanes 3 and 4: PCR with phosphorothioated–phosphorylated forward primer. Lanes 5 and 6: PCR products using normal forward primer (no modifications). Lane 7 and 8: PCR with phosphorylated forward primer. All PCRs have been done in the presence of 1 μM of forward primers and 0.02 μM of template. *B*, real-time monitoring of modified PCR primers using quantitative PCR technique. Primers used are as indicated in the legend within the figure. PC and NC are positive and negative controls, respectively.

A gradient PCR was performed with normal primers (suggested *T*_*m*_ of 61 °C) using a range of temperatures from 58 to 68 °C to find the best annealing temperature for a sharp PCR band. The sharpness of PCR product bands improved by increasing the annealing temperature, and it is at 68 °C that we obtained the best band (data not shown). Thereafter, PCR using modified primers was performed at 68 °C using a phosphorylated reverse primer, as well as (i) phosphorothioated, (ii) phosphorylated–phosphorothioated, (iii) normal, and (iv) normal-phosphorylated forward primers (Fig. 2*A*). As can be seen from Figure 2*A*, applying phosphorylation and phosphorothioate bond modifications did not have any effects on the quality of PCR bands, including for the PCR using a phosphorylated reverse primer and a protected (*i.e.*, phosphorothioated) and phosphorylated forward primer, the focus of this study. Furthermore, the performance of PCRs using modified primers was evaluated by quantitative PCR (qPCR) (Fig. 2*B*). Although signal intensity was decreased by ∼25% using modified phosphorylated and phosphorylated–phosphorothioated forward primers, the PCR product quantity is still high enough for all required applications. The quality of PCR products with one distinct length of dsDNA is important to get high yield and pure linear ssDNA preparation through exonuclease treatment and subsequently for circular DNA formation (34). Thus, optimization of PCR conditions, such as primers and template concentrations, number of cycles, and also prudent design of primers to avoid primer dimer formation, as well as ensuring specific binding of primers to template, is important to avoid pervasive effects on obtaining a defined double-stranded PCR band (35).

### Phosphorothioate protects 5′-phosphorylated DNA from degradation

**Figure 3:**
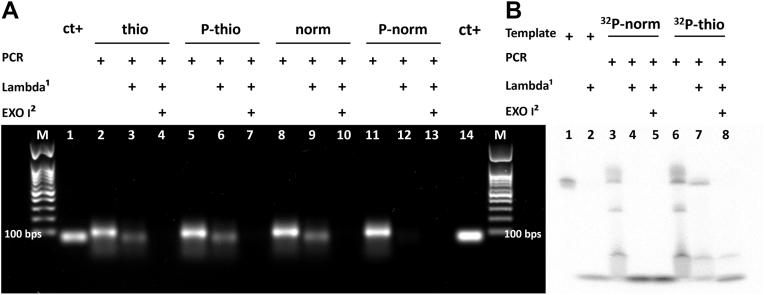
**Preparation of ssPCRP.***A*, visualization *via* a 2% agarose gel. Lane M: markers. Lanes 1 and 14: single-stranded amplicon with a final length of 100 bp (80 μM). Lanes 2, 5, 8, and 11: PCR products. Lanes 3, 6, 9, and 12: ssPCRP preparation with lambda exonuclease enzyme from PCR products. Lanes 4, 7, 10, and 13: ssPCRP treatment using exonuclease I enzyme. All PCRs were performed using phosphorylated reverse primers (1 μM). Lanes 2, 3, and 4; 5, 6, and 7; 8, 9, and 10; and 11, 12, and 13 have been done using phosphorothioated, phosphorothioated–phosphorylated, normal, and phosphorylated forward primers, respectively, as indicated in the figure, in a final concentration of 1 μM. *B*, ssPCRP analysis using 6% denaturing PAGE. Lanes 1 and 2: ^32^P-labeled 100-base oligonucleotide (0.1 pmol), intact or treated with lambda exonuclease, respectively. Lanes 3 and 6: PCR with phosphorylated reverse primers (1 μM). Lanes 4 and 7: ssPCRP preparation by lambda exonuclease. Lanes 5 and 8: ssPCRP incubation with exonuclease I enzyme. Lanes 3, 4, and 5; and lanes 6, 7, and 8: PCRs were done by employing normal-^32^P-labeled and phosphorothioated-^32^P-labeled forward primers, respectively, in a final amount of 4 pmol. ssPCRP, single-stranded PCR product.

DNA amplification through PCR generates dsDNA products (38). As previously mentioned, the lambda exonuclease enzyme has an exodeoxyribonuclease activity on dsDNA substrates from 5′ to 3′ and is about 20 times more active on a 5′-phosphorylated terminus compared with a nonphosphorylated one. Thus, by phosphorylating one of the primers of the PCR, it is possible to use the lambda exonuclease to easily get a single-stranded PCR product (ssPCRP) from a PCR product (35, 39, 40). In this work, to be able to use lambda exonuclease digestion reactions, we evaluated several forward primers with different chemical modifications combined with a phosphorylated reverse primer (to serve as substrate for digestion reactions). All PCR products (size of 100 base pairs) are aligned with the 100 base pair mark of the DNA ladders (Fig. 3*A*: lanes 2, 5, 8, and 11, as well as lane M). Whereas ssPCRPs (Fig. 3*A*: lanes 3, 6, and 9) ran lower in the gel and at the same level as the single-stranded 100-base template (Fig. 3*A*: lanes 1 and 14) confirming that ssPCRPs became single stranded after lambda exonuclease treatment. Moreover, to confirm that the lambda exonuclease–treated PCR products have been converted to ssDNA, we degraded the DNA with exonuclease I, a phosphodiesterase enzyme that degrades linear ssDNA in the 3′ to 5′ polarity (41, 42, 43). Since all ssPCRPs were digested by exonuclease I (Fig. 3*A*: lanes 4, 7, and 10), it confirmed the single-stranded nature of ssPCRPs. In addition, because of its 3′ to 5′ exodeoxyribonuclease activity, exonuclease I was not blocked by phosphorothioate bonds since these modifications are located at the 5′ extremities of the sense strands (36, 37). In principle, using a phosphorylated reverse primer would allow the production of ssPCRP after lambda exonuclease digestion; however, using a phosphorylated forward primer leads to both strands being degraded (Fig. 3*A*: lane 12). To obtain ssPCRPs, most laboratories ensure that the forward strand is protected by using a 5′ hydroxyl-forward primer (Fig. 3*A*: lane 9). Unfortunately, this is not compatible with applications that require a phosphorylated 5′ end. Although PCR sense strands without any modifications and with phosphorothioate bond modification were protected from degradation (Fig. 3*A*: lanes 6 and 9), it is noticeable that the phosphorylated sense strand with five phosphorothioate-modified bonds was protected from digestion by lambda enzyme (Fig. 3*A*: lane 6 and Fig. 1*C*). Lambda exonuclease action inhibition by phosphorylated–phosphorothioated modifications enables us to circularize the ssPCRP using a complementary strand without phosphorylation of the ssPCRP after the exonuclease reaction. Thus, in addition to excluding the phosphorylation step after exonuclease action, with this approach, the protected phosphorous group can be used for ssPCRP circularization by ligase enzymes (43, 44). Therefore, the whole circularization procedure has become much simpler and more efficient.

To verify the inhibition of lambda exonuclease action at the very 5′ phosphorylated end of PCR strands modified by phosphorothioated bonds, highly sensitive [γ-^32^P] radiolabeling was used. Phosphor imaging of lambda exonuclease degradation pattern of [γ-^32^P]-radiolabeled PCR products was evaluated using a phosphorylated reverse primer and [γ-^32^P]-radiolabeled forward primers without and with phosphorothioate modifications (Fig. 3*B*: lanes 3 and 6, respectively) and employing denaturing 6% PAGE (Fig. 3*B*). The two different undigested PCR products showed similar band patterns, with the main PCR bands migrating to the same level as the 100-base template (Fig. 3*B*: lanes 3, 6, and 1). Importantly, the normal sense PCR strand was completely digested by lambda exonuclease (Fig. 3*B*: lane 4), whereas the phosphorothioated sense PCR strand remained intact during the degradation reaction (Fig. 3: lane 7). This indeed verifies the effect of phosphorothioate modification to protect the strand of interest from degradation even if it has 5′ phosphate modification. Afterward, the radiolabeled-phosphorothioated ssPCRP was again digested by exonuclease I enzyme to confirm the single-stranded property of phosphorothioated ssPCRPs (Fig. 3*B*: lane 8). Finally, the [γ-^32^P] nucleotide residues resulting from digestion accumulated at the bottom of the gel, further verifying the digestion activity of exonuclease enzymes.

### ssPCRP circularization

**Figure 4:**
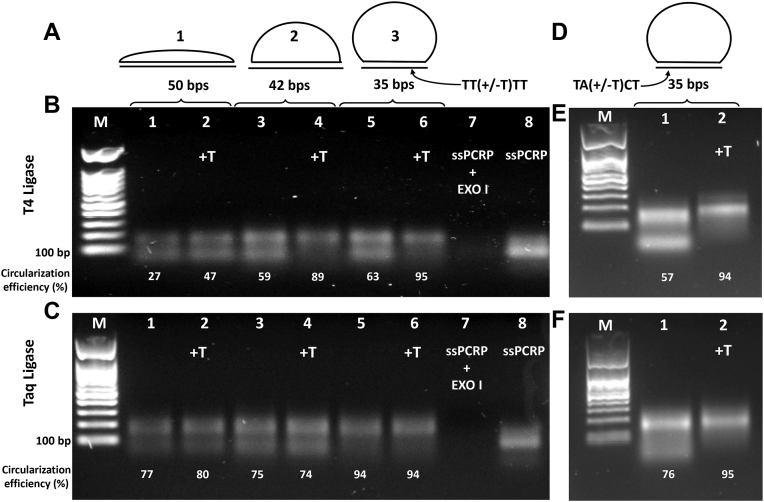
**ssPCRP circularization using complementary strands.***A*, Schematic representation of ssPCRP circularization with 50, 42, and 35 complementary bases bridging oligonucleotide containing TT(±T)TT at the ligation site. *B*, ssPCRP circularization with T4 DNA ligase. Lane M: marker. Lanes 1, 3, and 5: circularization reaction in the presence of 50, 42, and 35 bp complementary strands (TTTT sequences at ligation sites) and lanes 2, 4, and 6 in combination with 50, 42, and 35 bp complementary strands with an additional T nucleotide at the ligation site (TTTTT) at a final concentration of 1 μM, respectively. Lane 7: ssPCRP treatment with exonuclease I. Lane 8: ssPCRP. *C*, ssPCRP head-to-tail sealing using Taq DNA ligase enzyme. The order of the lanes is the same as *A*. *D*, schematic illustration of ssPCRP circularization using 35 bases of complementary strand–containing specific sequences at the ligation site. *E* and *F*, ssPCRP circularization *via* T4 and Taq DNA ligase enzymes, respectively. Lane M: marker; Lanes 1 and 2: circularization reactions using bridging oligonucleotides containing TACT or TATCT (additional T) sequence at the ligation position, respectively. ssPCRP, single-stranded PCR product.

Circular DNA has some advantages over linear DNA, including higher stability against misfolding and exonucleases as well as compatibility to be utilized as a template for exponential signal generation (5, 45). To convert linear ssPCRP to circular ssPCRP, six different complementary strands in different sizes, including 50, 42, and 35 bases, were employed as bridging oligonucleotides for ligation (Fig. 4*A*). As a matter of fact, Taq DNA polymerase enzymes add an additional adenosine nucleotide at the 3′ ends of PCR strands during amplification reaction (46, 47, 48). Thus, one additional thymine (T) nucleotide was embedded in the complementary strands exactly at the ligation spot to compare the ligation efficacy with thymine-less counterparts. Furthermore, in an attempt to improve the efficiency of ligation, two different DNA ligase enzymes were used: the T4 DNA ligase and the thermostable Taq DNA ligase (NEB) enzymes (Fig. 4, *B* and *C*). The ligation efficiency was affected by the presence of an additional T, the length of complementary strand, and the type of ligase enzyme. Since covalently closed circular ssPCRPs have lower migration rates as compared with linear ssPCRPs in agarose or PAGE gels (3, 49), we used gel electrophoresis to monitor ligation efficacy. As previously mentioned, the overall size of ssPCRP is 100 bases; thus, if a bridging oligonucleotide of 50 complementary bases is utilized for ssPCRP circularization, it hybridizes with half the length of ssPCRP, which likely hinders the full hybridization because of the lack of flexibility allowed by an ssDNA region with the same length as the dsDNA region (Fig. 4*A*, 1). However, by decreasing the complementary strand sizes, fewer bases in ssPCRPs are occupied in base pairing, which favors more efficient hybridization reactions (Fig. 4*A*, 1 and 2). When T4 DNA ligase and complementary strands with an additional T are used for ligation reactions, elevated ligation efficiency was seen for 42 and 35 base complementary strands compared with 50 bases for ssPCRP circularization (Fig. 4*B*: lanes 1, 2, 4, and 6). However, when complementary strands without additional T were used, we witnessed lower ligation efficacy even in the presence of 42 and 35 base strands (Fig. 4*B*: lanes 3 and 5), reducing by ∼30% the amount of circularized DNA; and, perhaps more importantly, increasing by as much as ∼7-fold the amount of linear DNA left over (from 5% to 37% for 35 bp bridging oligonucleotide). This can be explained by the adenosine nucleotide added by Taq DNA polymerase enzyme, which leads to a one-base mismatch pairing at the ligation site. Therefore, in the absence of the added T to the bridging oligonucleotide, T4 DNA ligase is not able to properly seal head to tail of the ssPCRP efficiently because of the base mismatch at this strategic spot (43, 50, 51). On the other hand, when ligation is done *via* the Taq DNA ligase enzyme, satisfying ligation bands were detected just by employing 35 base complementary strands even without an additional T (Fig 4*C*: lanes 5 and 6). Better ligation efficiency was expected *via* Taq DNA ligase because of its thermostability and ability to apply several denaturing–annealing cycles for ligation reaction (52). Although we observed lower selectivity for Taq ligase than T4 ligase enzyme at the ligation spot, for the purpose of circularization of a library, this does not represent a significant problem. Indeed, by using four T nucleotides at the ligation junction, we likely increased the ability of the DNA strand to be ligated even when using only four instead of the ideal five T nucleotides complementary to the extra A nucleotide added during PCR at the ligation junction (Table S1). Therefore, we assayed ligation efficiency in the presence of different bases at the ligation site (Fig. 4, *E* and *F*). Better ligation efficiencies were obtained for both T4 and Taq DNA ligase enzymes when bridging strands with an additional T are used. In addition, better signal intensity was observed by hyperbranched RCA (HRCA) reaction on circularized ssPCRP using +T bridging strand, which shows higher ssPCRP circularization rates compared with the strand without the additional T (Fig. S1). All in all, T4 DNA ligase would be a better candidate for ssPCRP circularization in this study because of its efficiency (90–95% of DNA ligated with bridging oligonucleotide of 42 and 35 bp), and the fact that it works at moderate temperature and indeed its lower price that can eventually improve the throughput of approaches that use the proposed method; overall providing a more user-friendly technique (53).

It is also worth noting that the formation of a 3′-5′ phosphorothioate linkage between the 3′-hydroxyl and the 5′-phosphorothioate of ssDNA can be catalyzed by ligase enzymes when the sulfur atom is involved only as a side group to the phosphorus and is not as a part of the bridging atoms (*i.e.*, 3′C–O–P–O–5′C) (54). In addition, phosphorothioate linkages are chiral at the phosphorus center, which results in two distinct diastereomers, namely Rp and Sp. According to the reported literature, only the Rp isomer is efficiently distinguished and ligated by ligase enzymes. As a result, this stereospecificity can significantly impact overall ligation efficiency in reactions involving phosphorothioate-modified oligonucleotides. Therefore, the configuration of the phosphorothioate group at the 5′ end of the ssDNA is important to ensure efficient ligation reaction and consequently successful circular DNA formation (55, 56).

### HRCA reaction using circularized ssPCRPs

**Figure 5:**
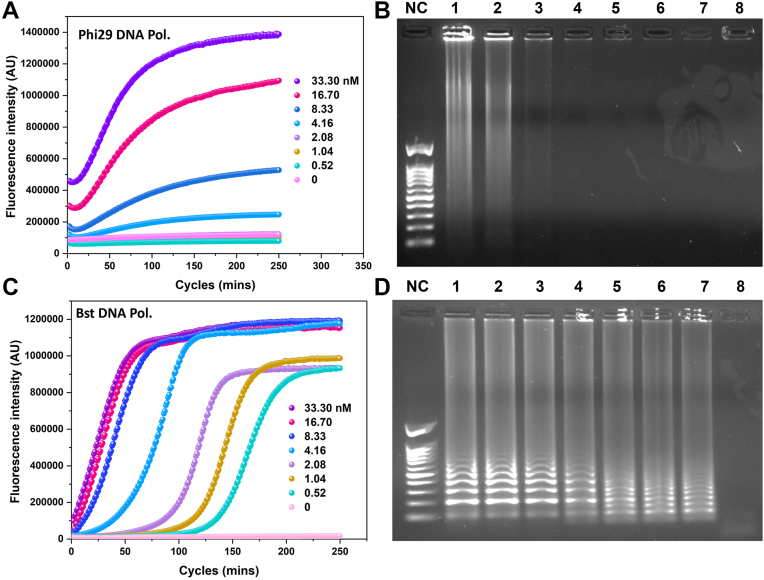
**HRCA reaction using circular ssPCRP.***A*–*C*, real-time monitoring of HRCA reaction *via* real-time PCR machine in different concentrations of circular ssPCRP (from *top* to *bottom*: 33.3, 16.7, 8.33, 4.16, 2.08, 1.04, 0.52, and 0 nM). *B*–*D*, visualization of HRCA product by agarose gel electrophoresis. Lane M: marker. Lanes 1 to 7: HRCA product from the highest concentration of circular ssPCRP (33.3 nM) to the lowest one (0.52 nM). Lane 8: negative control (no circular ssPCRP). HRCA, hyperbranched rolling circle amplification; ssPCRP, single-stranded PCR product.

To confirm that ssPCRP is covalently closed though a ligation reaction, circular ssPCRPs were used as a circular DNA template in the presence of PCR primers (forward and reverse) and phi29/Bst DNA polymerases for exponential HRCA amplification. Signal amplification caused by HRCA reaction leads to production of a large amount of dsDNA, which in turn could be monitored in real time by an intercalating fluorescent molecule (SYBR Green) (57). Fluorescence intensities increased overtime during exponential HRCA and reached plateaus when the amplification reactions were saturated (Fig. 5, *A* and *C*). Moreover, the intensities of fluorescence signal were proportional to the initial concentration of circular ssPCRP as observed from serial dilutions of circular ssPCRP (Fig. 5, *A* and *C*, from *top* to *bottom*). The products of real-time HRCA reaction were migrated on agarose gel to confirm production of high molecular weight and hyperbranched nature of the generated DNA, which lead to long smeared DNA bands (Fig 5, *B* and *D*: lanes 1–8: from high to low concentrations). It is also worth noting that better amplification rate and sensitivity was obtained by Bst DNA polymerase compared with the phi29 DNA polymerase because of its robustness in performing HRCA reaction (58, 59, 60, 61).

To assess whether the proposed method can be extended to longer ssPCRPs, 300 and 1000 base double-stranded PCR products were generated using the primers and targets listed in Table S1. As shown in both the agarose and PAGE gels (Fig. S5), the corresponding linear ssPCRPs were successfully prepared and validated (Fig. S5*A*: lanes 2 and 6). Circularization of the 300 and 1000 base ssPCRPs was performed using 41-nt bridging oligonucleotides, comprising 20 bases complementary on each side plus an additional T to pair with the overhanging A nucleotide (Fig. S5*B*: lanes 2, 3, 8, and 9). Successful HRCA products further confirmed effective amplification from the circular ssPCRP templates for both lengths (Fig. S5*A*: lanes 3, 4, 7, and 8; Fig. S5*B*: lanes 5, 6, 11, and 12).

### SELEX based on phosphorylated–phosphorothioated primers

To verify the performance of the proposed method in a relevant application, this technique has been used to select circular and linear aptamers through 15 successive rounds of SELEX against the whole MERS-CoV spike protein as well as a fragment from spike protein, the receptor-binding domain (RBD). In addition, four negative selections were performed using agarose magnetic beads and lysozyme protein immobilized on agarose magnetic beads to exclude nonspecific aptamers from the library (Fig. S2). By increasing SELEX rounds, the amount of ligand and ligand–library incubation time were gradually decreased, and washing times and number of washings were gradually increased. Therefore, based on the utilized conditions, selected aptamers through generation 15 are supposed to have a lower *K*_*D*_ compared with other cycles. In Table 1, selected aptamers were categorized based on their cluster sizes obtained from generation 15 and frequency of mutations that appeared during SELEX cycles. These results confirm the applicability of the proposed technique to generate diverse libraries for the SELEX procedure.

**Table 1:** Selected circular and linear aptamers

Target	Library	Reads/cluster (%)	Mutations (positions and frequencies)	*K*_*D*_ (nM)	Aptamer sequence (random region)
Spike	Linear	7.4	G2 (1.1%)	—	CATGGTGGAGACATAAAGCTTGAGAGGGTTCCAT
		2.4	G1 (6.2%)	—	A**CCGGCGTCACCAC**A**CGGGGAGGTTAGATGTGCCCAGGGTCC**T**GT**
		11.5	A1 (6%), C7 (1%)	—	G**CCGGCGTCACCAC**G**CGGGGAGGTTAGATGTGCCCAGGGTCC**C**GT**
		6.9	A1 (4.5%)	—	G**CCGGCGTCACCAC**G**CGGGGAGGTTAGATGTGCCCAGGGTCC**T**GT**
		5.1		—	**CCTAG**C**T**TGTGAAA--------GATCCACAGACCGCTGCTTGTTAGGGCTGGGTTCCC--
		23.9	T9 (8%), C10 (2%)	2.8	**CCTAGAT**GCTAC----------GGTTCAAAGCAGTGCT**TG**C**GCT**AAGGCTGGTGTACCTT
		19.4	C11 (1.15%)	—	**CCTAGAT**GCATTCAACCCGTTTGGTCTCAACTGTTGAA**TG**G**GCT**GGGTCC----------
	Circular	50	G6 (1%)	5	---**G**G**T**GAAC-TTGTTAG**TGT**A**GG**CTG**G**TGTACCTACACTA**TG**GCCCTA**G**ACAT
		34.5	C1 (4.5%)	—	TGC**G**A**T**TAGG-GTTTGGC**TGT**G**GG**CCT**G**GCAGAGATCGCAA**TG**GGGACG**G**G---
		5.4		—	GCC**G**G**T**GGTATTCACAGC**TGT**T**GG**TTT**G**GTTGGTCTGGTCT**TG**TGAGTT**G**----
RBD	Linear	0.9		—	CCGGGGGGGTGGGTGGGTAATGGCTGCGAATGCCTT**G**CCTTGTGAC----CCGA------
		59.5	G42 (1.5%)		**GCGCGCGAAAGTGACAATTG**---------**GGCCTCGGCTGTGATTG**T**TCGAGGGGAGAC**-
		2.3		—	**GCGCGCGAAAGTGACAATTG**---------**GGCCTCGGCTGTGATTG**C**TCGAGGGGAGAC**-
		12.5	T1 (3.3%), C2 (1%), C13 (2.8%), C29 (1%)	—	--CTGGGACTGCGATCTGTA**CACCTATC**TTTCCCAA**GATTAGAGACT**ACGGC--------
		6.6		—	-----------GCTGCAGTT**CACCTATC**CGTTTATG**GATTAGAGACT**TCTCGGGGACTAC
	Circular	45.5	C1 (4.5%)		TGC----**G**ATTAGGGTTTGG**C**T**GT**G**GG**CC**TGG**CA**G**AGA**T**CGCAATGG**G**GACGGG
		28.3	C44 (9%), A46 (13.5%)	—	--**GCCG-GTGGTATTCACAGCTGTTGGTTTGGTTGGTCTGGTCTTGTGTGT**TG-
		1.2	C48 (1%), T49 (2.3%)	—	--**GCCG-GTGGTATTCACAGCTGTTGGTTTGGTTGGTCTGGTCTTGTGTGT**CG-
		1.3		—	T-GCCGT**G**TCATGTCATCAC**C**A**GTTGG**A**TTGG**GG**GGTCTGG**CATTCT**G**GTG---

Different properties of selected circular and linear aptamers analyzed by AptaSUITE program. Samples from generation 15 were sent for sequencing (MiSeq), total reads obtained for each library is shown, as well as the number of reads corresponding to each selected sequence (reads/cluster). For the highest ranking aptamer sequences, mutations found in >1% of sequences are also shown. Other sequences with more than 1000 reads are also shown. The total number of reads for spike (linear and circular) and RBD (linear and circular) are 114,552, 129,423, 115,739, and 133,535, respectively. The bolded sequences in the aptamer sequence part are aligned using Clustal Omega software. The conserved sequences are underlined.

**Figure 6:**
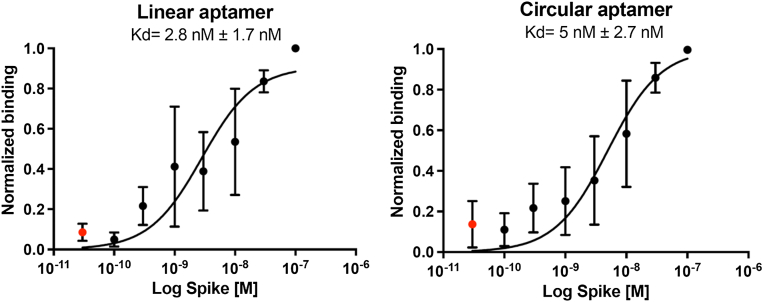
***K*_*D*_ determination with filter assays.** Binding affinity of the two selected aptamers (linear and circular selected with full spike). Average of results obtained by triplicate assays. Full spike (0.1–100 nM) in the presence of ∼0.5 nM of radiolabeled aptamers. *Red circles* corresponding to 0 nM spike (and not 3 × 10^−11^ as represented here) were added to the graphs to visualize the plateau.

After 15 successive cycles of SELEX, the circular and linear aptamers against full-size spike protein that ranked first (*i.e.*, with the most reads following sequencing) were selected for *K*_*D*_ determination. To do this, the linear and circular aptamers were radiolabeled with [γ-^32^P] using [γ-^32^P] ATP and T4 polynucleotide kinase (PNK), and the circular aptamer was circularized using the aforementioned methods. The radiolabeled linear and circular aptamers were then used in a filtration assay using a Bio-Dot apparatus in triplicate (Figs. 6 and S3) to obtain *K*_*D*_s in the nanomolar range for the full spike protein. Aptamers selected against RBD alone did not show binding when assayed with full MERS-CoV spike (data not shown). We also attempted to evaluate *K*_*D*_ by microscale thermophoresis using a complementary Cy5-labeled oligonucleotide, but the *K*_*D*_s obtained suggest that base pairing with the aptamer (even if it occurs in the constant region) interferes with aptamer–ligand interaction, as the estimated affinity is orders of magnitude weaker than the one obtained with the more direct filter assay. The numbers are nevertheless provided in Table 1 (more details in supporting information, Fig. S4).

## Discussion

As compared with other reported *in vitro* ssDNA generating techniques in the literature, the proposed method is more user friendly and has improvements as well as other advantages (Table 2) (3, 20, 29, 35, 62, 63). This method is much simpler and faster than conventional gel-based ssDNA preparation because the whole reaction happens in just a tube, which bypasses the time-consuming steps of gel preparation and running, as well as DNA elution and precipitation that are followed in gel-based procedures (31). In addition, ssDNA generation through biotin–streptavidin and NaOH treatment causes possible disruption of hydrogen bonds and hydrophobic forces between streptavidin and biotin, which leads to detachment of biotinylated strand and rehybridization with complementary strands in the solution and thus, the presence of unwanted dsDNA in the ssDNA preparation (32, 33). The same dsDNA impurity also happens in asymmetric PCR, where some level of DNA complementary to the strand of interest remains (19, 29). Lambda exonuclease digestion of a phosphorylated reverse strand bypasses the problems mentioned above (35) but is not compatible with applications that require a phosphate group at the 5′ end of the DNA strand. However, embedding chemical phosphorothioate modifications at the 5′ end inhibits the lambda exonuclease enzyme activity leaving the strand intact after the degradation of the reverse strand (32, 39). Therefore, in this study, we devised a 5′ phosphorylated strand that is not affected by the digestion reaction by also including phosphorothioate modifications. Our approach allows the omission of the normally required phosphorylation step before ssPCRP circularization, thus making the whole procedure simpler (4). Furthermore, in comparison to circle-to-circle amplification (C2CA), which is another enzymatical way for single-stranded linear or circular DNA production (64, 65, 66), the proposed method is faster and less complicated, and no dsDNA impurity is produced during the process. Indeed, in C2CA, to reach the sequence with desired polarity, it is necessary to do at least two rounds of C2CA reaction and repeat even more rounds to get more products (65). C2CA also has more dsDNA impurities because it includes steps of polymerization and endonuclease reactions where the produced ssDNA is partially double stranded (64).

**Table 2:** Comparison of different aspects of reported methods in the literature with the proposed technique

Method	Application	Type of modifications	Amplification method(s)	Gel-based technique	dsDNA impurity	Circular DNA preparation	Reference
Lambda exonuclease enzyme	ssDNA preparation	Phosphorothioate & phosphorylation	PCR	−	−	+	Present work
	ssDNA preparation	Phosphorylation	PCR	−	−	−	(35)
Biotin–streptavidin	SNP detection	Biotinylation	PCR	−	+	+	(3)
	Genomic DNA production	Biotinylation & phosphorylation	PCR & RCA	−	+	+	(20)
	ssDNA preparation	Biotinylation	PCR	+	+	−	(62)
C2CA	Gene detection	−	RCA	−	+	+	(65)
Denaturing PAGE	Aptamer selection	Hexaethyleneglycol & fluorescein	PCR	+	−	−	(70)
Asymmetric PCR	ssDNA preparation	Phosphorylation	PCR	−	+	−a	(29)

a This study has not performed circularization with the ssDNA, but it would theoretically be possible.

By employing a template with random sequences and the nonproofreading Taq DNA polymerase enzyme, as well as DNA circularization optimization using a complementary strand and DNA ligase enzymes, we confirmed the applicability of this method for either linear or circular aptamer selection through the SELEX procedure. Furthermore, because the method does not rely on gels, and the whole reactions are done in a tube, it is possible to automate ssDNA preparation using this procedure. This technique is a good alternative for coupling of PCR with HRCA for exponential gene generation and for PCR-based PLPs, as well as single-stranded library production in general (2, 3, 19, 20, 21). Sequencing technologies such as Nanopore and PacBio use circularized DNA templates to increase the quality of sequencing (67, 68, 69); the method explained herein could provide an alternative to the current protocols, which could, in addition, also allow ligation of a few DNA fragments before circularization, thus providing a longer fragment for sequencing of even longer reads compared with the current methodology. Moreover, it was recently found that long ssDNA provides a very efficient way of making CRISPR-mediated knockins (23); in this case also, the inclusion of a phosphate at the 5′ end of PCR products allows ligation of different ssPCRP to make ssDNA beyond the typical range of PCR products, allowing insertions of larger DNA fragments in the genome, in addition to simplifying the procedure for ssDNA preparation (or reducing the cost if megamers are used). Overall, the proposed method is likely to help many technical advancements in a number of areas in molecular biology and genetics.

## Experimental procedures

### DNA library, oligonucleotides, and primers

The 100-base template with two primer binding sites (25 bases for each) and 50 random bases in the middle is provided from Integrated DNA Technologies. All primers (forward and reverse) either with modifications (phosphorothioate bonds and phosphorylation) or without modifications and aptamers were purchased from Sigma–Aldrich Company. Complementary strands bridging oligonucleotides were obtained from Integrated DNA Technologies and Alpha ADN company (Table S1).

### PCR and ssPCRP preparation

PCR amplification was performed in PCR mixture including template (0.02 μM), phosphorylated reverse primer (1 μM), phosphorothioated–phosphorylated forward primer (1 μM), 0.20 μM dNTP (DGel Electrosystem), Milli-Q water, 10X HotStarTaq buffer (QIAGEN), and 0.5 units HotStarTaq DNA Polymerase (QIAGEN) in final volume of 50 μl. PCR was achieved in 12 cycles and at the optimized melting temperature (*T*_*m*_) of 68.2 °C in a C1000 touch thermal cycler (Bio-Rad). For ssPCRP preparation, each 10 μl of double-stranded PCR products were treated with 5 units of lambda exonuclease enzyme (New England Biolabs [NEB]) at 37 °C for 45 min, which is followed by 10 min at 75 °C for enzyme inactivation.

### ssPCRP circularization through ligation reaction of phosphorothioated DNA

Circularization of ssPCRP requires the phosphorothioated–phosphorylated products because products that are merely phosphorylated (which is necessary for ligation by ligase enzymes) will be completely degraded by the lambda exonuclease enzyme that is used to make ssDNA (see the previous section). The phosphorothioated positions protect the phosphorylated DNA strand chosen to make circular DNA.

To circularize ssPCRP, ligation reactions with T4 DNA ligase were prepared: 1 μM of ssPCRP was added to 1 μM complementary strands in 1X T4 ligation buffer (NEB) in a final volume of 10 μl. For the circularization reaction, DNA was denatured by increasing temperature to 95 °C for 5 min and then gradually cooled down to room temperature for DNA hybridization and circular ssPCRP formation. Afterward, the ligation reaction was performed by introducing 10 units of T4 DNA ligase enzyme (NEB) and incubating at 20 °C for 1 h to seal the 5′ and 3′ ends of the ssPCRP. Alternatively, it is possible to use the Taq DNA ligase with ∼20 cycles of denaturation at 95 °C for 1 min followed by annealing and ligation steps using proper *T*_*m*_ for 1 min (*T*_*m*_ of 68, 64, and 60 °C for 50, 42, or 35 complementary bases between strands, respectively). After ligation, either by using T4 or Taq DNA ligase enzymes, 10 units of exonuclease I enzyme (NEB) are added to ligation mixture for noncircular ssPCRP degradation.

### HRCA reaction

HRCA reaction was performed using different dilutions of circular ssPCRP (33.33, 16.66, 8.33, 4.16, 2.08, 1.04, 0.52, and 0 nM) in a final volume of 30 μl of amplification mixture including 1X phi29 DNA polymerase/Bst 2.0 WarmStart DNA Polymerase buffers (NEB), 0.3 μM nonmodified PCR forward and reverse primers, 0.40 μM dNTP, Milli-Q water, SYBR Green 1X (Invitrogen), 10 μg bovine serum albumin, and 8 units of phi29 DNA polymerase/Bst WarmStart DNA Polymerase enzymes (NEB). Amplification reactions were monitored for 5 h at 30 °C or 65 °C (for phi29 or Bst DNA polymerases, respectively) in MicroAmp fast reaction tubes with cap (Applied Biosystems) and using real-time PCR machine (Applied Biosystems).

### Agarose gel electrophoresis

Agarose gel electrophoresis has been conducted in 2% agarose gel and 1X TAE buffer (1 mM EDTA and 40 mM Tris–acetate; Fisher Scientific) and by applying constant voltage of 120 V for 40 to 60 min. Thereafter, gels were illuminated under UV radiation *via* gel doc (Bio-Rad) and 1X preadded gel stain (TransGen Biotech). Power supply and DNA ladder were purchased from Bio-Rad and Bio-Helix, respectively. Bands were quantified with ImageJ (Wayne Rasband, NIH). To determine circularization ratios, the intensity of the band corresponding to circular DNA was divided by the sum of both bands corresponding to circular and linear DNAs (a blank value was subtracted for each band).

### [γ-^32^P] radiolabeling

[γ-^32^P] labeling of 100-base template and forward primers (phosphorothioated and nonphosphorothioated) was manipulated in a final volume of 20 μl labeling mixture containing 1X T4 PNK enzyme buffer (NEB), Milli-Q water, 20 pmol of DNA substrate, 5 μCi [γ-^32^P] ATP (Revvity), and 10 units of T4 PNK enzyme at 37 °C for 30 min.

### Visualization *via* PAGE

Phosphorimaging of [γ-^32^P]-radiolabeled DNA sequences was accomplished through 6% denaturing PAGE constituted of 1X TBE (89 mM Tris base, 2 mM EDTA, and 89 mM boric acid; Fisher Scientific) (57), 8 M urea (BioShop), 6% acrylamide/*bis*-acrylamide (BioShop), 16 μl *N*,*N*,*N*′,*N*′-tetramethylethane-1,2-diamine (Fisher Scientific), 300 μl ammonium persulfate 10% (BioShop) in a total volume of 40 ml. Denaturing PAGE ran for 1 h under constant power of 15 W. Subsequently, the gel was exposed to a phosphorimager cassette (GE Healthcare) for 10 min, and finally, the cassette was scanned by a Typhoon FLA 9500 biomolecular imager (GE Healthcare).

### Spike protein immobilization

Recombinant MERS-CoV spike protein and MERS-CoV spike protein fragment were produced from the MERS-CoV genome sequences (human betacoronavirus 2c EMC/2012), which encodes spike protein and RBD, respectively. These recombinant MERS-CoV spike proteins were fused with a polyhistidine tag at the C terminus and expressed in Baculovirus insect cells (Sino Biological).

Recombinant spike protein immobilization on agarose magnetic beads (cyanogen bromide–activated SepFast MAG 4HF; BioToolomics) was performed based on the protocol provided by BioToolomics. In this work, 10 mg of each recombinant protein was immobilized on 300 mg/ml agarose magnetic beads. After doing several steps for spike protein functionalization on agarose magnetic beads, the immobilized beads were washed several times using SELEX buffer and held using a magnet to remove the unbound spike proteins from stock solution. Thereafter, immobilized spike protein was diluted in 500 μl of SELEX buffer, which was used as stock spike protein solution to perform 15 cycles of SELEX. This solution should be vortexed well before addition to each cycle to make sure that homogeneous concentration of immobilized protein is added to SELEX cycles.

### SELEX procedure

Circular library preparation was performed in final T4 ligation mixture of 100 μl including 1X T4 DNA ligase buffer (NEB), 1 μM phosphorylated library, 1 μM 35 bp complementary strand, 20 U T4 DNA ligase enzyme (NEB), and milliQ water for 1 h at 16 °C, which was followed by enzyme inactivation for 20 min at 80 °C. Thereafter, the ligation reaction was treated by 10 U of exonuclease I enzymes (NEB) for 1 h at 37 °C for degradation of noncircularized library. Then, the exonuclease was inactivated at 80 °C for 20 min.

The first round of SELEX was started separately in the presence of 100 pmol of linear and circular libraries using immobilized spike proteins and spike protein fragments. During the first round, the linear and circular libraries were incubated for 2 h with immobilized spike proteins on agarose magnetic beads at room temperature in 1 ml of 1× SELEX buffer (20 mM Tris–HCl [pH 7.4], 2 mM CaCl_2_, 5 mM KCl, 100 mM NaCl, and 1 mM MgCl_2_). Thereafter, the bound libraries were collected by applying a magnet (Invitrogen), which is followed by supernatant removal and washing agarose beads with 500 μl 1× SELEX buffer. Afterward, the beads were diluted in 20 μl of SELEX buffer, and 5 μl of the diluted library was directly used for enrichment step using PCR protocol described in the main text of the article and in the final volume of 50 μl. In this step, a phosphorothioated forward primer and a phosphorylated reverse primer were used for the linear library, and for the circular library, a phosphorothioated–phosphorylated forward primer and a phosphorylated reverse primer were utilized. After verification of PCR bands using agarose gel, ssPCRPs were produced from PCR products using the previously described procedure, and at this point, the linear library has been regenerated for another round of SELEX. For the circular library, the produced phosphorylated libraries were circularized using the process explained earlier with a T4 DNA ligase enzyme for sealing the library head to tail. The positive selection process has been done for 15 rounds of SELEX using circular and linear libraries and using the spike protein and the RBD as targets. Moreover, four SELEX rounds were performed as negative selection cycles using nonspecific targets including agarose magnetic beads and lysozyme protein immobilized on agarose magnetic beads (Table S2). From SELEX rounds 1 to 15, concentration of ligands and incubation time have gradually decreased, whereas number of washes as well as washing time have gradually increased. The detailed information about SELEX conditions used for each SELEX round is shown in Table S2.

### Radiolabeled circular and linear aptamer preparation and *K*_*D*_ determination using filter assay

Radiolabeling of linear and circular aptamers, as well as circularization of 5′ phosphorylated aptamers, was done as described above and using 35 bp complementary strand without the additional T. The radiolabeled circular and linear aptamers against full spike were then purified from 6% denaturing PAGE by cutting the circular or linear bands, the latter running further in the gel than the former as explained and eluted using elution buffer (0.3 M NaCl) at 4 °C overnight. The aptamers were precipitated using a precipitation solution (0.1 volume 3 M NaCl and 2.2 volume 95% ethanol) at −80 °C and washed two times using 70% ethanol. The dried pellets were finally solubilized in Milli-Q water. Thereafter, radiolabeled circular and linear aptamers were incubated for 1 h at a final concentration of 0.5 nM with the full spike using increasing concentrations (0.1, 0.3, 1, 3, 10, 30, and 100 nM) in 1× SELEX buffer in a final volume of 50 μl; the negative control used (0 nM) is 1× SELEX buffer. A Bio-Dot Apparatus (Bio-Rad) was used for the filter assay with the following membranes, from bottom to top: two Whatman filter papers, one Hybond-N+ membrane (Amersham), and one nitrocellulose membrane (Bio-Rad). The two membranes were soaked in Milli-Q water and then in 1× SELEX buffer for 20 min beforehand. The filter assay was done by loading the Bio-Dot wells with 1× SELEX buffer two times, and then the aptamer-spike solutions were loaded and aspirated by vacuum. The membranes were exposed to a phosphor imager cassette and scanned by the Typhoon FLA 9500. The *K*_*D*_ was determined by normalizing the intensity of the radioactive aptamers bound to increasing concentrations of full spike (Hybond-N+) to the free DNA (nitrocellulose) and quantified using the software ImageJ. Graphs and *K*_*D*_ were done using Graph Pad (Graph Pad Software, Inc).

### Quantitative PCR

Real-time monitoring of qPCR using modified primers, including normal, phosphorylated, phosphorothioated, and phosphorylated–phosphorothioated forward primers as well as a phosphorylated reverse primer, was done in mixtures including 1X reaction mixture of GoTaq 2-Step RT–qPCR kit (Promega), 400 nM forward and reverse primers, and in a final volume of 20 μl. PCR was performed a total of 20 cycles and at an annealing temperature of 68 °C and *via* an Applied Biosystems qPCR machine.

## Data availability

All data are contained within the article.

## Supporting information

This article contains supporting information.

## Conflict of interest

The authors declare that they have no conflicts of interest with the contents of this article.
