# Supplementary material for: Simple in vitro single-stranded linear and circular DNA preparation, functional selection, and validation using phosphor-derived modifications
Source: J Biol Chem. 2025 Oct 29;301(12):110874. doi: 10.1016/j.jbc.2025.110874 (PMC12721166; doi:10.1016/j.jbc.2025.110874)
Supplement: Supplementary materials [file mmc1.docx]

**Supplementary materials for:**

Simple in vitro single stranded linear and circular DNA preparation, functional selection and validation using phosphor-derived modifications

Seyed Vahid Hamidi^1,2^, Vanessa Aguilar-Sánchez^1^, Vincent Héroux^1^ and Jonathan Perreault^1^*

^1^INRS Centre Armand-Frappier Santé Biotechnologie, 531 boulevards des Prairies Laval (QC) Canada.

^2^now at Department of Bioengineering, McGill University, Montreal, Quebec, Canada

Email: [jonathan.perreault@inrs.ca](mailto:jonathan.perreault@inrs.ca)

Table S1 Oligonucleotide sequences used in this study.

| PCR and RCA forward primers | 5′-AACGCCTATGTCTCGTTGAAGCAGC-3′ |
| --- | --- |
|  | 5′-P-AACGCCTATGTCTCGTTGAAGCAGC-3′ |
|  | 5′-A*A*C*G*C*CTATGTCTCGTTGAAGCAGC-3′ |
|  | 5′-P-A*A*C*G*C*CTATGTCTCGTTGAAGCAGC-3′ |
|  | 5′-P-T*A*C*G*C*CTATGTCTCGTTGAAGCAGC-3′ |
| 300/1000 bp | 5′-P-C*G*C*G*G*AACCCCTATTTG-3′ |
| PCR and RCA reverse primer | 5′-P-TTGCCGGTGACAGACTGCTTGCATA-3′ |
|  | 5′-P-CTGCCGGTGACAGACTGCTTGCATA-3′ |
| 300 bp | 5′-P-TGGAAAACGTTCTTCGGG-3′ |
| 1000 bp | 5′-P-TCTAAAGTATATATGAGTAAACTTGGTCTG-3′ |
| Template/library for linear and circular aptamers | 5′-AACGCCTATGTCTCGTTGAAGCAGCNNNNNNNNNNNNNNNNNNNNNNNNNNNNNNNNNNNNNNNNNNNNNNNNNNTATGCAAGCAGTCTGTCACCGGCAA-3′ |
|  | 5′-P-AACGCCTATGTCTCGTTGAAGCAGCNNNNNNNNNNNNNNNNNNNNNNNNNNNNNNNNNNNNNNNNNNNNNNNNNNTATGCAAGCAGTCTGTCACCGGCAA-3′ |
| PCRp/300bp AmpR/pET15b | 5′-CGCGGAACCCCTATTTGTTTATTTTTCTAAATACATTCAAATATGTATCCGCTCATGAGACAATAACCCTGATAAATGCTTCAATAATATTGAAAAAGGAAGAGTATGAGTATTCAACATTTCCGTGTCGCCCTTATTCCCTTTTTTGCGGCATTTTGCCTTCCTGTTTTTGCTCACCCAGAAACGCTGGTGAAAGTAAAAGATGCTGAAGATCAGTTGGGTGCACGAGTGGGTTACATCGAACTGGATCTCAACAGCGGTAAGATCCTTGAGAGTTTTCGCCCCGAAGAACGTTTTCCA-3′ |
| PCRp/1000bp AmpR/pET15b | 5′-CGCGGAACCCCTATTTGTTTATTTTTCTAAATACATTCAAATATGTATCCGCTCATGAGACAATAACCCTGATAAATGCTTCAATAATATTGAAAAAGGAAGAGTATGAGTATTCAACATTTCCGTGTCGCCCTTATTCCCTTTTTTGCGGCATTTTGCCTTCCTGTTTTTGCTCACCCAGAAACGCTGGTGAAAGTAAAAGATGCTGAAGATCAGTTGGGTGCACGAGTGGGTTACATCGAACTGGATCTCAACAGCGGTAAGATCCTTGAGAGTTTTCGCCCCGAAGAACGTTTTCCAATGATGAGCACTTTTAAAGTTCTGCTATGTGGCGCGGTATTATCCCGTGTTGACGCCGGGCAAGAGCAACTCGGTCGCCGCATACACTATTCTCAGAATGACTTGGTTGAGTACTCACCAGTCACAGAAAAGCATCTTACGGATGGCATGACAGTAAGAGAATTATGCAGTGCTGCCATAACCATGAGTGATAACACTGCGGCCAACTTACTTCTGACAACGATCGGAGGACCGAAGGAGCTAACCGCTTTTTTGCACAACATGGGGGATCATGTAACTCGCCTTGATCGTTGGGAACCGGAGCTGAATGAAGCCATACCAAACGACGAGCGTGACACCACGATGCCTGCAGCAATGGCAACAACGTTGCGCAAACTATTAACTGGCGAACTACTTACTCTAGCTTCCCGGCAACAATTAATAGACTGGATGGAGGCGGATAAAGTTGCAGGACCACTTCTGCGCTCGGCCCTTCCGGCTGGCTGGTTTATTGCTGATAAATCTGGAGCCGGTGAGCGTGGGTCTCGCGGTATCATTGCAGCACTGGGGCCAGATGGTAAGCCCTCCCGTATCGTAGTTATCTACACGACGGGGAGTCAGGCAACTATGGATGAACGAAATAGACAGATCGCTGAGATAGGTGCCTCACTGATTAAGCATTGGTAACTGTCAGACCAAGTTTACTCATATATACTTTAGA-3′ |
| Complementary bridging strands | 5′-GCTGCTTCAACGAGACATAGGCGTTTTGCCGGTGACAGACTGCTTGCATA-3′ |
|  | 5′-GCTTCAACGAGACATAGGCGTTTTGCCGGTGACAGACTGCTT-3′ |
|  | 5′-CAACGAGACATAGGCGTTTTGCCGGTGACAGACTG-3′ |
|  | 5′-CAACGAGACATAGGCGTACTGCCGGTGACAGACTG-3′ |
| Complementary bridging strands with additional T | 5′-GCTGCTTCAACGAGACATAGGCGTTTTTGCCGGTGACAGACTGCTTGCATA-3′ |
|  | 5′-GCTTCAACGAGACATAGGCGTTTTTGCCGGTGACAGACTGCTT-3′ |
|  | 5′-CAACGAGACATAGGCGTTTTTGCCGGTGACAGACTG-3′ |
|  | 5′-CAACGAGACATAGGCGTATCTGCCGGTGACAGACTG-3′ |
| 300 bp | 5′-AAACAAATAGGGGTTCCGCGTTGGAAAACGTTCTTCGGGGC-3′ |
| 1000 bp | 5’-AAACAAATAGGGGTTCCGCGTTCTAAAGTATATATGAGTAA-3’ |
| Labeled  complementary  strand | Cy5-ACATAGGCGTT |

P indicates phosphorylation modifications

*shows phosphorothioate bond modifications


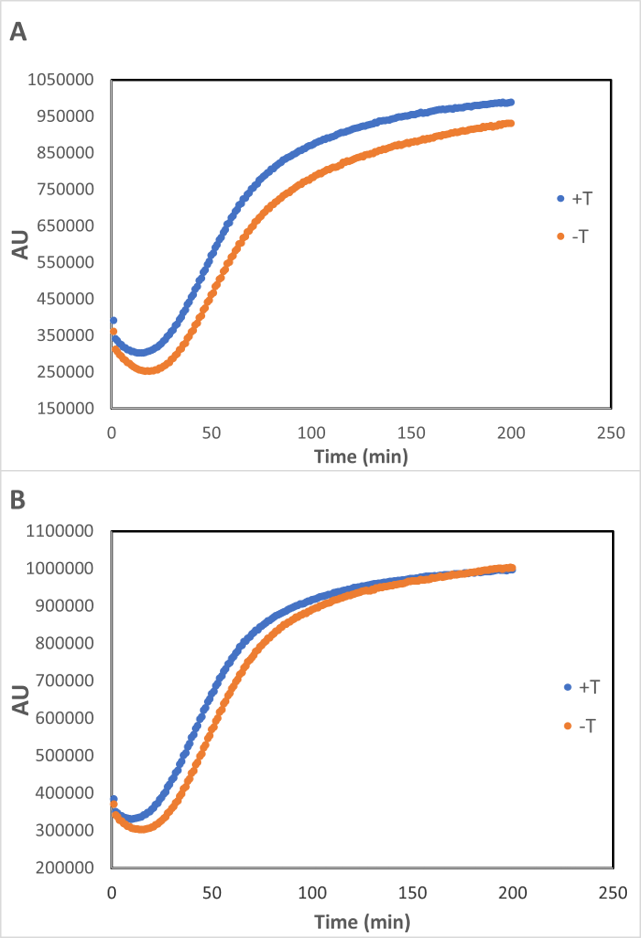


Figure S1 HRCA reaction using circularized ssPCRP with and without additional T. (A) Monitoring of HRCA reaction via circularized ssPCRP sealed by T4 DNA ligase using specific complementary strand. (B) HRCA reaction through circular ssPCRP sealed by Taq DNA ligase and specific complementary strand.


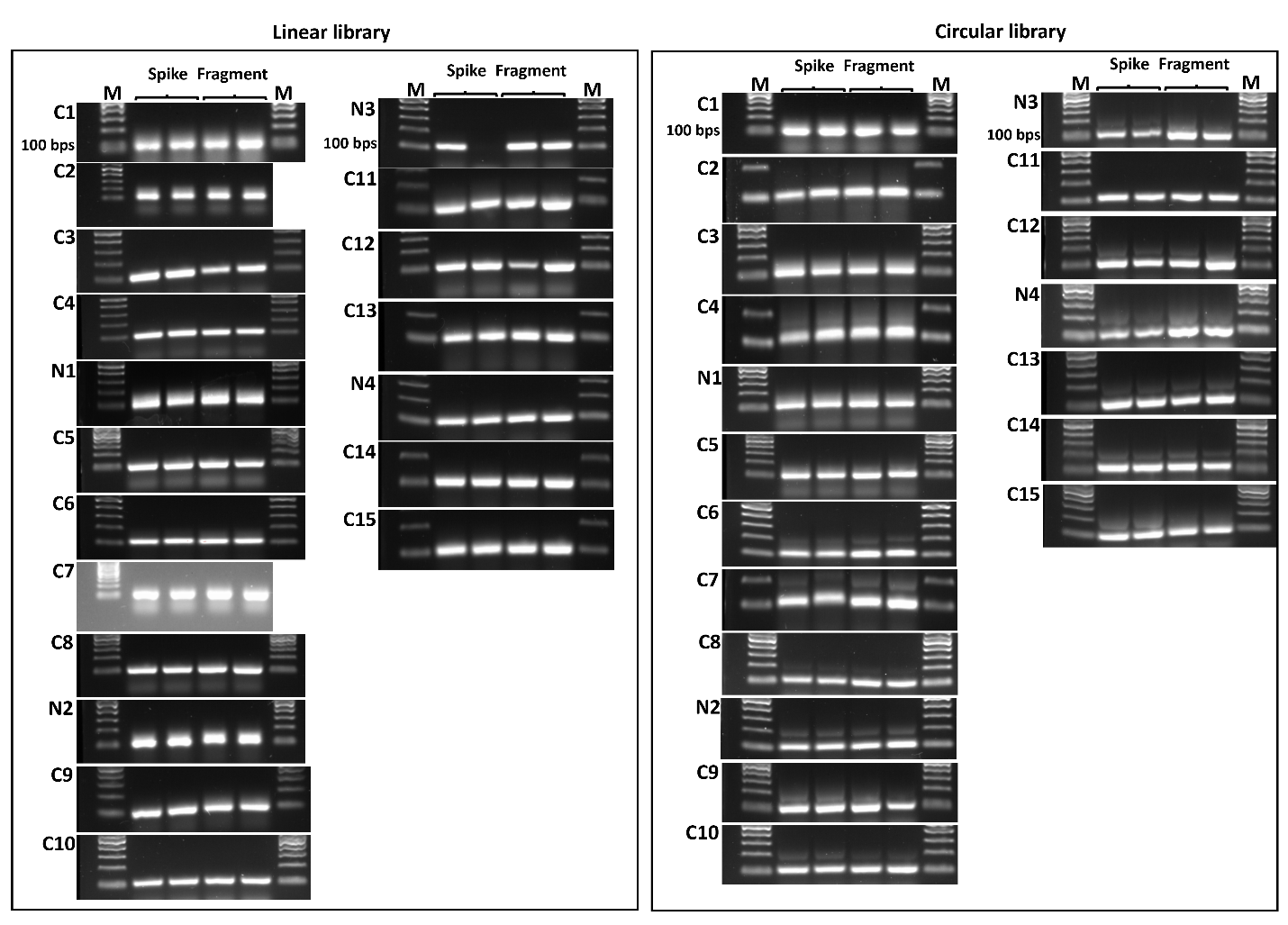


Figure S2 Amplification of selected libraries after each cycle of SELEX for linear and circular aptamer selection targeting MERS-CoV spike protein. M, C and N refer to molecular weight markers, SELEX cycle, and negative selection respectively. The reason why additional bands are seen in PCR amplification products of circular libraries, especially generations 6, 7, N2 is that it is likely that two ssPCRs were hybridized with two complementary strands during ligation process and thus dimers were produced during ssPCRs circularization reaction. PCR products are shown in duplicate for each full spike or RBD because during each SELEX round, one PCR product was used for the next SELEX round and the other one was kept as a backup in case something went wrong during the SELEX process, both are shown.

Table S2 Detailed conditions used for circular and linear aptamer selection against MERS CoV spike protein.

| Target | Library | Rounds | Incubation time (min) | No of washes | Added target  (µl) | Washing time  (min) |
| --- | --- | --- | --- | --- | --- | --- |
| Full spike  & fragment (RBD) | Linear & circular | C1 | 120 | 1 | 200 | 30 |
|  |  | C2 | 120 | 1 | 20 | 30 |
|  |  | C3 | 120 | 1 | 20 | 30 |
|  |  | C4 | 120 | 1 | 20 | 30 |
|  |  | N1 | 30 | - | 10 (beads) | - |
|  |  | C5 | 105 | 1 | 20 | 30 |
|  |  | C6 | 90 | 1 | 20 | 30 |
|  |  | C7 | 75 | 1 | 20 | 45 |
|  |  | C8 | 60 | 1 | 20 | 45 |
|  |  | N2 | 60 | - | 15 (beads) | - |
|  |  | C9 | 45 | 2 | 20 | 45 |
|  |  | C10 | 30 | 2 | 15 | 45 |
|  |  | N3 | 30 | - | 10 (lysozyme) | - |
|  |  | C11 | 30 | 3 | 15 | 60 |
|  |  | C12 | 30 | 2 | 10 | 60 |
|  |  | C13 | 30 | 3 | 10 | 60 |
|  |  | N4 | 60 | - | 15 (lysozyme) | - |
|  |  | C14 | 10 | 3 | 10 | 60 |
|  |  | C15 | 5 | 2 | 5 | 60 |


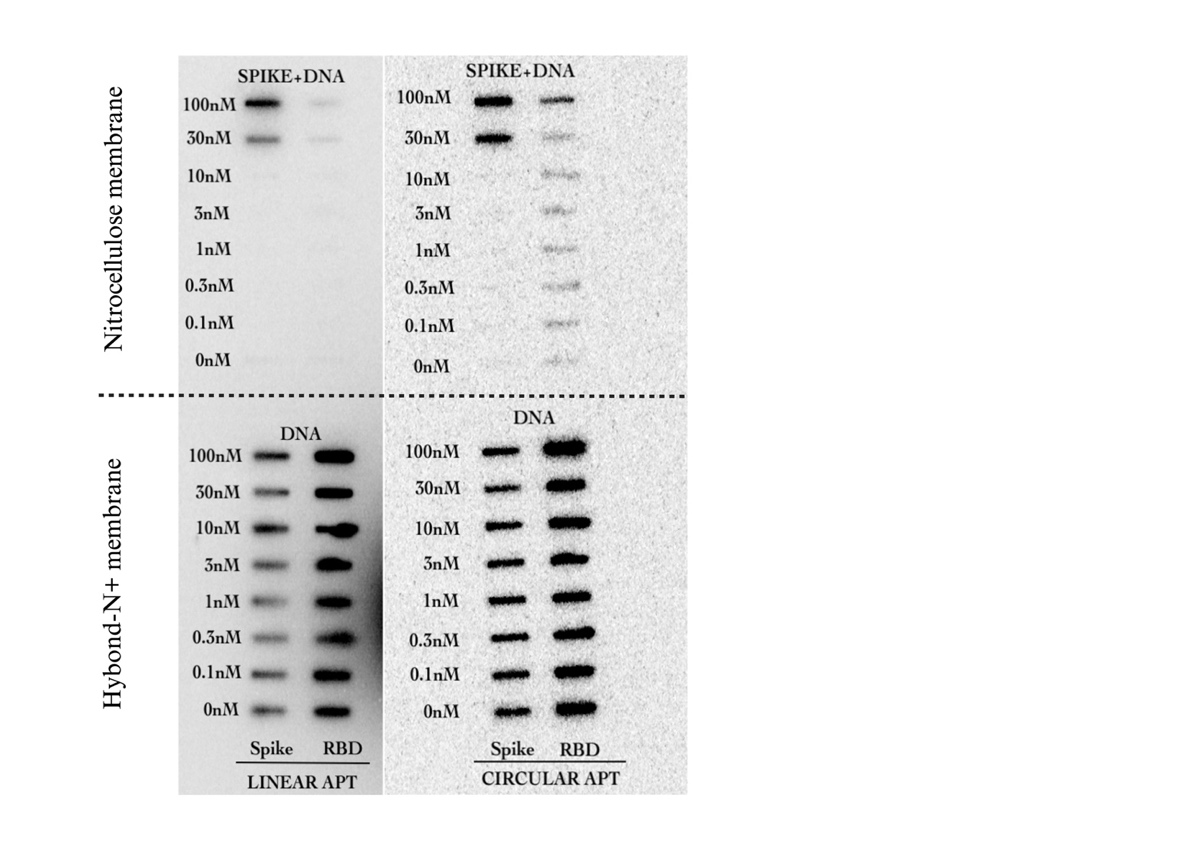


Figure S3 Filter assay. Linear and circular aptamers against full Spike and RBD (0.1 to 100 nM) in presence of ~0.5 nM of radio-labeled aptamers. The results for the aptamers against RBD barely show any binding for the protein (nitrocellulose membrane, where we observe low presence, or complete absence, of the aptamer signal; or background binding to the membrane in the case of the circular aptamer). Since the RBD-based selection may have led to aptamers incapable of binding the full spike protein, only results of aptamers identified from the SELEX against full spike are shown in the main text.


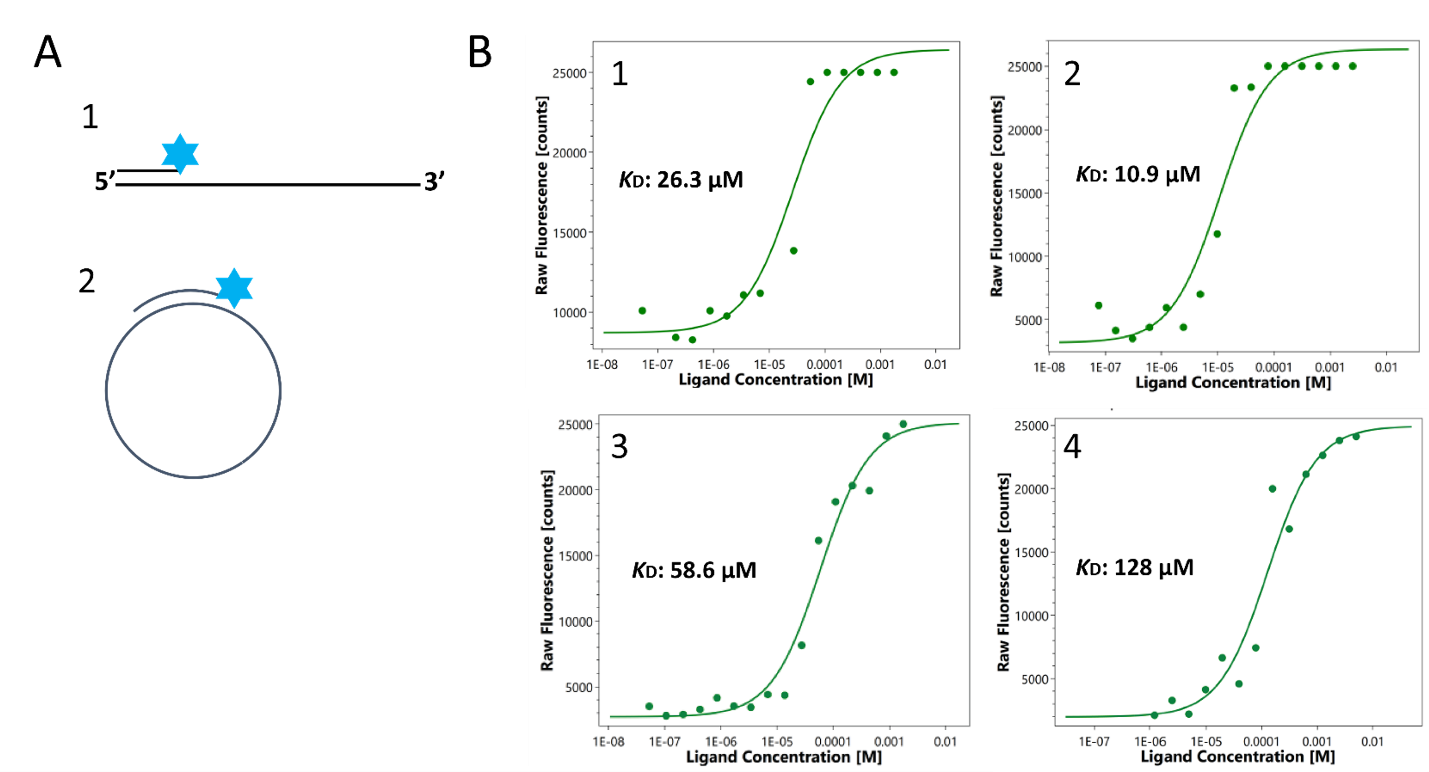


**Figure S4** KD determination. (A) Schematic illustration of selected linear and circular hybridization using labeled Cy5 complementary strand. (B) Binding affinity of selected aptamers (Linear/full spike (1), Linear/RBD (2), circular/full spike (3) and circular/RBD (4)) in the presence of 5 nM of labeled aptamers and 1mM to ~ 10^-8^ M of ligands (full spike and RBD).

*K*_D_ determination using microscale thermophoresis (MST) was performed via aptamers labeled with Cy5 and NanoTemper Monolith machine. Since the circular aptamers are covalently closed circular DNA and thus have no ends for 5' Cy5 modifications, a short labeled complementary strand which is specific to the 5' end region of aptamers were used (Fig. S4 and Table. S1). The same approach was employed for linear aptamers *K*_D_ determination to be able to compare affinities with circular aptamers (Fig. S4). As it can be seen from Fig. S4, an average *K*_D_ of ~ 50 µM was obtained using labeled complementary strand and MST. In addition, the selectivity of selected aptamers was also determined in the presence of bovine serum albumin (BSA) as a non-specific ligand and no specific binding was detected (data not shown). Since labeled short oligo strategy was used in this experiment, the selectivity of the procedure was determined in the presence of full spike and RBD and labeled complementary stands (without adding aptamers) and no affinity was observed (data not shown). This shows that the complementary strand has no affinity to the specific targets. Note that if it had a lesser impact on *K*_D_, such an MST-based assay could be useful to help design and characterize potential switching systems where binding of the cognate ligand might displace the oligonucleotide to provide a signal easy to adapt to other platforms.


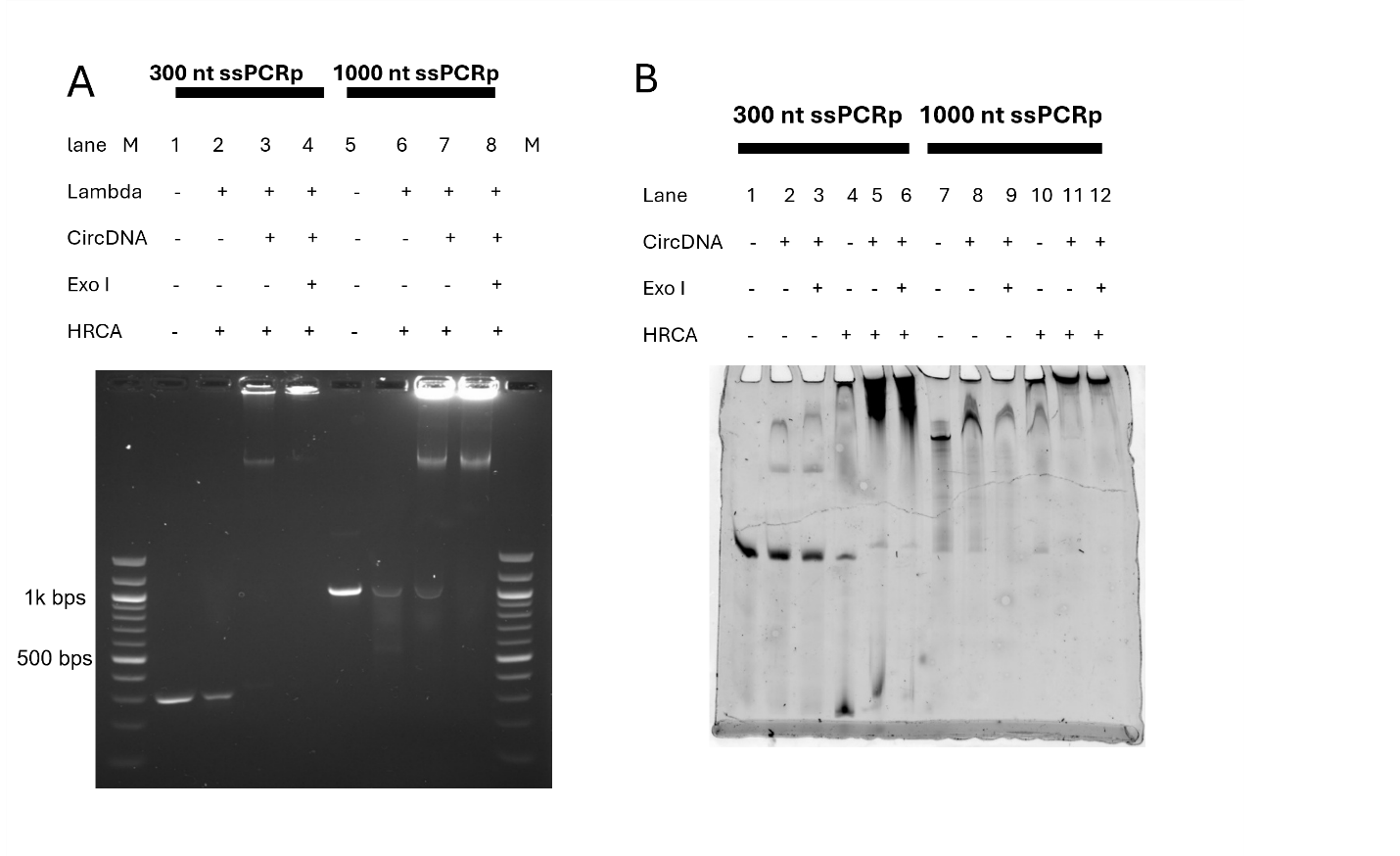


**Figure S5** Long circular ssPCRp preparation. (A) Long circular ssPCRp validation by agarose gel. Lane M: DNA molecular weight marker (100 bp ladder). Lane 1: PCR product for 300 bases ssPCRp preparation. Lane 2: HRCA reaction on linear 300 bases ssPCRp. Lanes 3 and 4: HRCA reaction on circular 300 bases ssPCRp (without and with Exonuclease I). Lane 5: PCR product for 1000 bases ssPCRp preparation. Lane 6: HRCA reaction on linear 1000 bases ssPCRp. Lane 7 and 8: HRCA amplification reaction on circular 1000 bases ssPCRp (without and with Exonuclease I). (B) Long circular library validation by 5% PAGE gel. Lane 1: 300 bases ssPCRp. Lane 2: circular ssPCRp preparation. Lane 3: 300 bases circular ssPCRp treatment with Exonuclease I. Lane 4: HRCA reaction on linear 300 bases ssPCRp. Lane 5 and 6: HRCA reaction on circular 300 bases ssPCRp (without and with Exonuclease I). Lane 7: 1000 bases ssPCRp preparation. Lane 8: 1000 bases circular ssPCRp preparation. Lane 9: 1000 bases circular ssPCRp treatment with Exonuclease I. Lane 10: HRCA reaction on linear 1000 bases ssPCRp. Lane 11 and 12: HRCA reaction on circular 1000 bases ssPCRp (without and with Exonuclease I).

**Experimental procedures:**

*Long ssPCRp of 300/1000 bases preparation, circularization and validation by HRCA*

All 300 and 1000 bp PCRp amplification oligonucleotides (5’ phosphorylated-phosphorothioated direct primer, 5’ phosphorylated reverse primers and 41 nt complementary bridging strands) were purchased from Galenvs Sciences. A PCR was performed using Taq DNA polymerase (FroggaBio) with the AmpR promoter and gene from a pET15b backbone (Table S1). The products were gel-purified using the QIAquick Gel Extraction Kit (Qiagen). Circularization was carried out using a method similar to ssPCRP circularization through ligation of phosphorothioated DNA. In detail, 168 ng of each purified PCRp was digested into ssDNA with 5 units of Lambda Exonuclease for 1 h at 37 °C. The reaction was supplemented with 1x T4 DNA ligase buffer and bridging oligos (900 and 270 fmoles for the 300 bp and 1000 bp PCRPs, respectively; ~1:1 ssPCRp:bridge), heated to 95 °C for 5 min, and gradually cooled down at room temperature. T4 DNA ligase was then added, and circularization was allowed to proceed for 1 h at 20 °C which was followed by heat inactivation at for 20 mins at 80 ˚C. Subsequently, 100 units of Exonuclease I were added for 30 min at 37 °C, followed by inactivation at 80 ˚C for 20 min. For HRCA, 5 µL of the circularization mix were added to the phi29 HRCA amplification mixture (as discussed previously) and 300/1000 bp PCR/RCA forward and reverse primers for 5 h at 37 °C, then heat-inactivated at 65 °C for 10 min. The products were analyzed on a 5% PAGE gel (one-third of the HRCA reaction and the remaining circularization mix), stained with ssGreen (Lumiprobe) for 40 min, and imaged with a Typhoon FLA 9500. In addition, two-thirds of the HRCA reaction were run on a 1% agarose gel (using 1X TBE buffer) alongside equal amounts of purified PCRp as a weight standard and imaged using a Bio-Rad Gel Doc system.
